# Time-space analysis of highly pathogenic avian influenza H5N2 outbreak in the US

**DOI:** 10.1186/s12985-016-0605-4

**Published:** 2016-08-30

**Authors:** Nutthawan Nonthabenjawan, Carol Cardona, Alongkorn Amonsin, Srinand Sreevatsan

**Affiliations:** 1 Center of Excellence for Emerging and Re-emerging Infectious Diseases in Animals, Faculty of Veterinary Science, Chulalongkorn University, Bangkok, Thailand; 2 Department of Veterinary Public Health, Faculty of Veterinary Science, Chulalongkorn University, Bangkok, Thailand; 3 Department of Veterinary Population Medicine, College of Veterinary Medicine, University of Minnesota, 1971 Commonwealth Avenue, Rm 301E, St. Paul, MN 55108 USA; 4 Department of Veterinary and Biomedical Sciences, College of Veterinary Medicine, University of Minnesota, 1971 Commonwealth Avenue, Rm 301E, St. Paul, MN 55108 USA

**Keywords:** Evolutionary, H5N2, Highly pathogenic avian influenza, Minnesota

## Abstract

**Background:**

In early 2015, highly pathogenic avian influenza H5N2 caused outbreaks in commercial poultry farms in Minnesota and neighboring states where more than 48 million birds were affected. To date, the origin and transmission pathways of HPAI H5N2 have not been conclusively established.

**Methods:**

In this study, we analyzed forty-six samples from turkeys and their environment that were collected at different time-points of the outbreak to identify origins and within outbreak evolutionary changes. We performed de-novo whole genome sequencing from primary samples and the most recent common ancestors of the PB2, PA, HA5, M and NS segments were traced back to Japanese HPAI H5N8 isolates. These segments appeared to have diverged from the ancestor around June and November 2014.

**Results:**

The time to most recent common ancestor analysis for PB1, NP and NA2 segments suggest two likely possibilities of reassortant HPAI H5N2 origin - either a reassortment in Alaska area or multiple reassortments with North American low pathogenic avian influenza strains, before the HPAI H5N2 outbreak strain emerged. Within the outbreak, viruses clustered into two and three subgroups suggesting high substitution rates of 0.702x10-2 - 1.665x10-2 (subs/site/year), over the 5-month outbreak period.

**Conclusions:**

Data are suggestive of a fast evolving HPAI strain within an outbreak that should be taken into consideration in developing appropriate control strategies in the future.

**Electronic supplementary material:**

The online version of this article (doi:10.1186/s12985-016-0605-4) contains supplementary material, which is available to authorized users.

## Background

Avian influenza virus (AIV) is an enveloped virus that contains eight negative sense single strand RNA segments which encode at least ten functional proteins [1]. Hemagglutinin (HA) and neuraminidase (NA) are surface proteins that are used to classify AIV into subtypes. At present, 16 HA and 9 NA subtypes have been identified in aquatic wild birds which are a natural reservoir of these viruses [2]. AIV can be divided into low pathogenic avian influenza viruses (LPAI) and highly pathogenic avian influenza viruses (HPAI) by its ability to cause disease in poultry which is identified by intravenous pathogenicity index (IVPI) test or by it’s possession of poly-basic amino acid feature at the HA cleavage site [3].

Outbreaks HPAI H5 in Guangdong, China in 1996 have likely origins from goose Guangdong lineage HPAI viruses [4]. Since 1997, HPAI H5N1 dispersed in more than 50 countries in Africa, Asia, Europe, and the Middle East. In 2003, a re-emergence of HPAI H5N1 in China and South East Asia was reported. Since its emergence, the H5 gene has continued to evolve and has been arbitrarily classified in the OIE nomenclature system into clades identified from 0 to 9 [5]. The latest clade, 2.3.4.4, was designated in January 2015 and replaced the provisional clade 2.3.4.6. The first isolation of H5 clade 2.3.4.4 viruses was from domestic mallard ducks (*Anas platyrhynchos*) in China in 2008 [6]. The H5 clade 2.3.4.4 has demonstrated an ability to reassort with multiple neuraminidase subtypes including N1, N2, N3, N5, N6 or N8 [7].

In January 2014, HPAI H5N8 caused outbreaks in 161 commercial poultry flocks in South Korea and led to culling of 14,000,000 birds [8]. In early November 2014, HPAI H5N8 was detected in a turkey flock in Germany followed by an outbreak in a duck farm in England and a chicken farm in the Netherlands. Wild birds may play an important role in generating novel reassortant subtypes of clade 2.3.4.4 and carrying viruses across continents [9]. In late November 2014, HPAI H5N2 caused outbreaks in turkey and broiler breeder flock in British Columbia, Canada. In the United States, the first case of HPAI H5 was detected in a captive gyrfalcon (*Falco rusticolus*) in Washington in early December and subsequently the first detection of the reassortant HPAI H5N2 was reported in a northern pintail duck (*Anas acuta*) in Washington. In December 2014 and January 2015, HPAI H5 viruses associated with various NA subtypes were detected in backyard poultry flocks in Oregon and Washington, USA [10]. In January 2015, an HPAI H5N8 virus was detected in a commercial turkey flock in California [11]. On March 2^nd^, 2015, the first case of HPAI H5N2 in the Midwestern USA was confirmed in Pope County, Minnesota. On June 17th, 2015, last confirmed case in this outbreak was reported in Iowa. In the Midwest region, more than 200 confirmed cases were reported and more than 48,000,000 birds were affected [12].

In this study, we investigated the origin and within outbreak evolution of this new emergent HPAI H5N2 that caused outbreaks in Midwestern states.

## Methods

### Sample collection

Samples were collected from turkeys and the environment at locations involved in outbreaks of HP clade 2.3.4.4 H5N2. Sampling was performed over a 106-day period during the outbreak. A total of 46 tissue or oropharyngeal or environmental samples were collected. Samples were divided into three phases representing an approximate 35-day interval each - early phase (March 4th–April 7th) [*n* = 18], mid phase (April 8th–May 12th) [*n* = 19] and late phase (May 13th–June 17th) [*n* = 9].

All samples were identified by RT-PCR testing water and bird samples from all barns of turkey flocks to identify infected samples per established methods [13]. All RT-PCR positive samples (water in infected barns, tracheal and cloacal swabs from birds or air) collected at early, mid and late phases of the outbreak were genome sequenced directly from primary samples (Table 1).

**Table 1:**
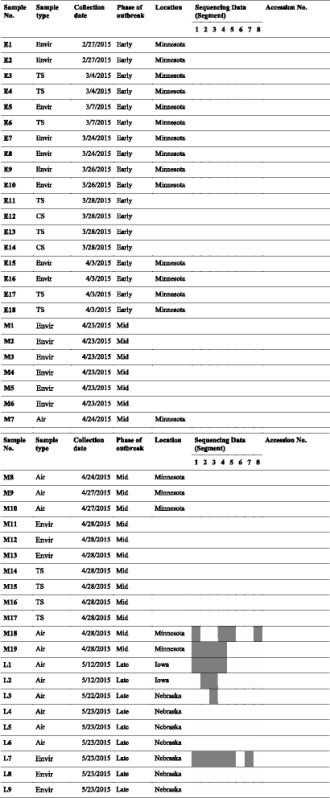
Details of samples in this study

Note: White and grey box indicates available and unavailable sequencing data on each segment, respectively. TS, CS and Envi stand for tracheal swab, cloacal swab and environmental sample, respectively

### Whole genome sequencing

Forty-six samples from turkeys, drinker biofilm, air and environment submitted to the University of Minnesota Mid Central Research and Outreach Center (Willmar, MN) were used in this study. RNA was extracted with the MagMAX™-96 Viral RNA Isolation Kit (Ambion) using a magnetic particle processor (Kingfisher, model 700) according to manufacturer’s instructions. All RNA samples were tested by RT-PCR for matrix gene as described [13]. The positive RNA samples were subjected to amplify all eight segments of virus simultaneously using a one-step RT-PCR as described [14]. Briefly, SuperScript® III one-Step RT-PCR system with Platinum® Taq DNA polymerase (Invitrogen™; CA, USA) was used with 1.6 mM and 0.2 μM final concentration of Magnesium and primers, respectively. PCR products were imaged by electrophoresis in 1.5 % agarose gel and subsequently purified by QIAquick PCR Purification Kit (Qiagen®; Hilden, Germany). Purified PCR products were submitted to University of Minnesota Genomics Center (UMGC) for illumina paired-end 250 cycles sequencing by using Nextera XT DNA kit for library generation. Sequences were assembled by mapping all reads to a reference and whole- or partial-genome sequences were extracted via CLC genomics workbench module available on Minnesota supercomputing institute (MSI) resources at the University of Minnesota.

### Phylogenetic analysis

The reference nucleotide sequences were obtained from Influenza Research Database (http://www.fludb.org/) and GISAID (http://gisaid.org/) in May 2016. Reference sequences were selected to represent previous and recent avian influenza strains from varying geographic areas including North America and Eurasia. The following approach was applied to select reference sequences: 1) include an ancestral strain of clade 2.3.4; 2) use of sequences of isolates selected from 3 well characterized H5 (2.3.4.4 lineage) outbreaks in Asia, Europe and North America; and 3) use of a double selection criteria to identify the top 30 hits to the current outbreak isolates by BLAST and develop a tree. Subsequently, these closely related sequences were reanalyzed by BLAST to expand the reference database and used to reconstruct phylogeny. LPAI North American strains that were isolated during 2012–2015 were also included. Reference and sample sequences from the current study were aligned using Muscle v.3.6 [15] subsequently any extra sequences beyond and after start and stop codon were trimmed. Maximum clade credibility (MCC) tree of each gene segments were generated by BEAST 1.8 with Bayesian Markov Chain Monte Carlo (BMCMC) algorithm. Strict clock model with coalescent constant population and HKY with gamma 4 substitution was used as model parameters [16, 17].

Mean substitution rate was estimated by Bayesian coalescent with constant population size [16] and strict clock model was applied. The Bayesian MCMC chain lengths were 10,000,000 generations with sampling every 10,000 generations and the effective sample size (ESS) value was assessed by using Tracer (v1.6.0) (Molecular evolution, phylogenetics and epidemiology, Edinburgh, Scotland, UK) [18]. Every gene segment analysis had ESS value greater than 200 to suggesting minimal standard error. The resulting tree of each iteration was summarized for a representative clustering pattern by using a tree annotator with 10 % discarding of the chains as burn-in and the resulting maximum clade credibility tree was visualized with FigTree software (v1.4.2) (Molecular evolution, phylogenetics and epidemiology, Edinburgh, Scotland, UK).

## Results

First case of this series of outbreaks in the Midwest area was confirmed on March 4th, 2015 in Pope county, Minnesota and last case on June 17th, 2015 in Iowa [12]. A 106-day period was divided into three phases representing an approximate 35-day interval - early phase (March 4th–April 7th), mid phase (April 8th–May 12th) and late phase (May 13th–June 17th). Eighteen, nineteen and nine samples were collected during early, mid and late phases of the outbreak, respectively (Table 1).

### Origin of HPAI H5N2

**Fig. 1:**
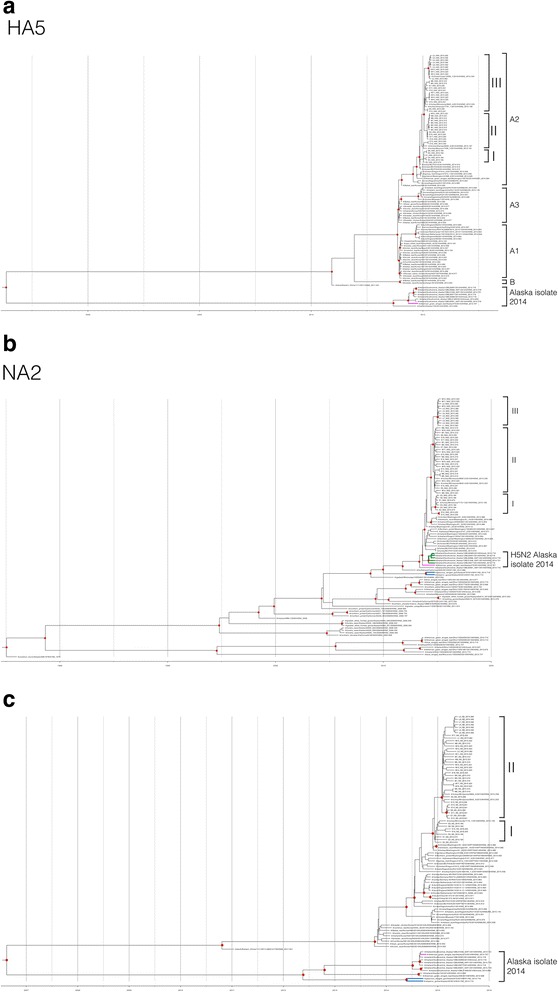
Time-scaled Bayesian maximum clade credibility tree inferred for the HA5 **a**, NA2 **b** and NS gene **c**. Trees were generated by Bayesian Markov Chain Monte Carlo algorithm in Bayesian evolutionary analysis by sampling trees. The TMRCAs representing the estimated timing of viral divergence from their ancestor are provided in parentheses. Red dot at each node represents the posterior probability above 0.7. A/AGWT/AK/472/14, group of A/mallard/SAK/14 and non-H5 LPAI NAm strain were labeled by pink, green and blue branch. Clustering is shown with reference strains of recent ancestry and within outbreak viruses clearly cluster in 2 or 3 clades separated by 35-day intervals. The TMRCA

Forty samples were successfully whole genome sequenced and six provided whole segment sequences of some gene segments as shown in Table 1. Phylogenetic analysis of HPAI H5N2 (HPAI H5N2 EA/NA) showed that it was a reassortant between Eurasian (EA) HPAI H5N8 and North American (NAm) LPAI. HPAI H5N2 EA/NA genetic constellation is composed of five gene segments (PB2, PA, HA, M and NS) from EA HPAI H5N8 and the remaining three segments (PB1, NP and NA) from NAm LPAI (Fig. 1a, b, c and Additional file 1a–e).

**Fig. 2:**
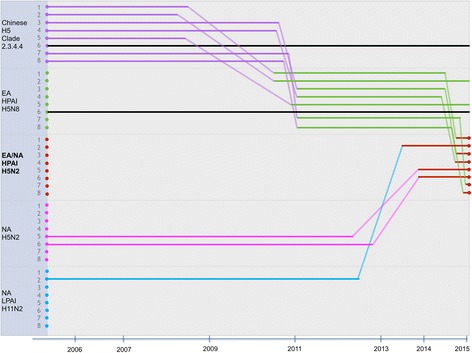
Reconstruction of the genetic constellation of reassortment events leading up to the emergence of EA/NA H5N2. The eight genomic segments are represented as parallel lines in descending order of segment 1 to 8. Each colored line represent transmission pathways of influenza genes from different ancestor: Chinese H5 Clade 2.3.4.4 (*purple*), Eurasian HPAI H5N8 (*green*), Eurasian/North American HPAI H5N2 (*red*), LPAI H5 North American strain (*pink*) and non-H5 LPAI North American strain (*blue*). Black line represents the different NA subtype that was not included in the analysis. Slopes lines represent divergent dates of each segment at 95 % highest posterior density interval

Time to most recent common ancestor (TMRCA) analysis showed that EA HPAI H5N8 likely evolved from an AIV H5 clade 2.3.4.4 (China) between April 2008 and January 2011 (2008.297–2010.255). Longer-term analysis of HPAI H5N2 EA/NAm, TMRCA analysis indicated that PB2, PA, HA5, M and NS segments diverged from EA HPAI H5N8 (Japan) strain, around June and November 2014 (2014.438–2014.900). PB1 was closely related to non-H5 LPAI North American strain isolated from Alaska (blue branch) likely diverged from these isolates around June 2012 and June 2013 (2012.453–2013.438, mean TMRCA 2012.950). NP appears to have diverged from A/American green-winged teal/Alaska/472/2014 (A/AGWT/AK/472/14; pink branch) an LPAI H5N2 North American strain, during September 2012 and December 2013 (2012.721–2013.941, mean TMRCA 2013.358). NA2 ancestry traced back to H5N2 that was isolated from Alaska during flu season 2014–2015 (pink and green branches) and reassortment likely occurred around December 2012 and November 2013 (2012.993–2013.824, mean TMRCA 2013.400). TMRCA of all gene segments are summarized in Fig. 2. The TMRCA format (E.g., 2006.272) was calculated using the formula: collection date divided by the number days in a year. For example, January 10 2006 is day 10^th^ of the year; TMRCA = (1/365)*10 = 0.027 in the year 2006 = 2006.027.

### Within outbreak evolution

The mean substitution rate of all segments over the 106-day interval of this study was estimated to be between 0.702 and 1.665 **×** 10^−2^. The NS gene segment was the most divergent segment while NP gene segment was the most conserved segment (Table 2). Bayesian Coalescent analysis showed that every gene segment of samples in this study formed three clades (I, II and III) except M and NS segment that form two major clades (I and II) (Fig. 1a–c and Additional file 1a–e). Members in subgroup I of PB2, PB1, PA, HA5, NP and NA2 were consistent in terms of clustering by time within the outbreak period with some minor variations. For example two samples (E15 and E16) fell into cluster II for M while the same samples clustered in clade I for NS. The HA5 segment of Korean H5N8 clustered within groups A and B [8]. Group A is a predominant cluster and appears to have dispersed to other continents. In addition, this cluster was subdivided into 3 clades (A1, A2 and A3) [19]. HPAI EA/NAm H5N2 belongs to the A2 subgroup which same subgroup as the two isolates H5N8 (Japan) and HPAI H5N2 (Canada) lineages (Fig. 1a). These results of high polymorphism rate and diversification of gene segments over the course of the epidemic indicate rapid evolution of viruses within this outbreak. These analyses assume evolution from a single introduction. Thus, that this high rate of change could likely be explained by multiple introductions cannot be ruled out.

**Table 2:** Mean nucleotide substitution rate of the H5N2 epidemic

	Mean Substitution rate (×10^−2^)	Substitution rate 95 % HPD (×10^−2^)
PB2	1.079	0.526–1.710
PB1	1.160	0.620–1.760
PA	0.712	0.332–1.150
HA	1.290	0.670–2.000
NP	0.702	0.261–1.240
NA	1.413	0.644–2.310
M	1.329	0.315–2.540
NS	1.665	0.595–2.790

### Genetic analysis for amino acid variability

Genetic analysis of the HA5 segment indicated multiple basic amino acid at HA cleavage site. Samples from early and mid phase of the outbreak contain the motif PLRERRRKR/GLF that is a characteristic of H5 clade 2.3.4, while samples collected during late phases of the outbreak carried the PQRERRRKR/GLF motif Glutamine (Q) is the major amino acid of other H5 clades [20]. Amino acid substitutions were analyzed by comparing with A/Northern pintail/Washington/40964/2014 (A/NP/WA/14), the first isolate of HPAI H5N2 EA/NA in the US. At position 141 (H5 numbering system) on the HA gene, A/NP/WA/14 and sub-cluster I contained serine (S) while 17 samples, sub-cluster II and III, contain proline (P). S141P mutations are related to surface protein stability and reduced host immune response [21]. The deduced amino acid sequence of the HA stalk region of all samples were identical.

All samples contained S at position 31 on M2 that has been shown to confer reduced susceptibility to amantadine and rimantadine antiviral drugs. However, analysis of resistance to neuraminidase inhibitors (NAI) at position 119, 151, 222, 224, 276, 292 and 371 on NA2 gene segment [22] showed that all samples in this study contain the conserved amino acid at every position which are expected to maintain NAI susceptibility. The E627K and D701N substitutions in the PB2 gene segment that are expected to increase virulence in mammals were examined. The results showed that all samples contain E and D in respective amino acid sites suggesting low mammalian virulence in this group of viruses.

## Discussion

A study addressing the spread of HPAI H5N8 from South Korea to Europe and Japan have suggested that these outbreaks originated from a single source population [23]. Dispersion of the virus to different geographic localities coincides with wild bird migratory seasons strongly suggesting their important role in the transmission of this pathogen and has been suggested as a mechanism by which H5N8 entered South Korea [24]. The most likely flyway involved in the recent spread of HPAI EA H5N8 to Japan was the East Asian Australian (EAA) Flyway [23]. The EAA flyway extends from north-eastern Asia and western Alaska to the southern extent of Australia and New Zealand and Mongolia, western China and eastern India [25]. On the North America continent, Alaska is an area where there is major overlap between migratory birds from the EAA and Pacific Flyways [25] but there exists no evidence of HP H5 clade 2.3.4.4 in Alaska. The existence of PB1, NP and NA2 ancestor that related to HP H5N2 in Alaska arise the possibility that HPAI H5N2 EA/NA originated in a location that coincides with Alaska and subsequently spread to British Columbia, Canada and Washington, USA. However, the mechanism and timing of viral transfer from the Pacific Flyway to the Mississippi Flyway in NA has not been established.

The ancestral strain and the overlapping of interval between TMRCA estimates of PB1, NP and NA2 suggest two hypothesis of HPAI EA H5N2 origin. First, it is possible that reassortant event occurred in Alaska. This finding is concordant with the report of avian influenza surveillance in Alaska during spring and summer 2015 that found reassortant LPAI between EA and NAm [26]. Second, the outbreak viral genome constellation likely occurred via multiple reassortment events with an LPAI before addition of genes of HPAI EA H5N2 origin due to the earlier divergence time of PB1, NP and NA2. Furthermore, HA5 clade 2.3.4.4 has the potential to reassort with multiple NA subtypes including NA1, NA2, NA5, NA6 and NA8 [27]. For example, reassortant H5N1 (EA/NA H5N1) containing PB2, HA5, NP and M from EA H5N8 and the rest of the gene segments (PB1, PA, NA1 and NS) from AIV North American lineage was detected in a green wing teal and an American wigeon in December 2014, with no subsequent case reports. This finding indicates that EA/NA H5N1 was likely unable to persist in the population while EA/NA H5N2 provided a compatible constellation for maintenance. Alternately, this may indicate that there was insufficient surveillance to detect the fate of that reassortment.

The nucleotide substitution rate of all segments within the outbreak are greater than 5.000 × 10^−3^ substitution/site/year which are higher than the rates estimated in long term analysis [28]. These results indicated that viruses were evolving rapidly during the current outbreak. Host jump from wild birds to domestic poultry including turkeys and chickens is a factor that provoked this rapid evolution. Therefore, identification of bridge of interspecies transmission is an important process to prevent the further outbreaks. While the HA gene had an extremely high substitution rate, the HA stalk region of all samples were identical at the amino acid level. The HA stalk region, PA and NP proteins were highly conserved, opening avenues for a universal subunit vaccine concept and development.

At present, no human case has been reported due to HPAI H5N2 EA/NA and it is considered as low health risk to public health [29]. However, three human cases with severe respiratory disease caused by HPAI H5N6 HA5 clade 2.3.4.4 have been reported in China [30]. These cases confirmed that HA5 clade 2.3.4.4 is capable of causing human infection and thus ongoing monitoring of viral evolution should be performed.

## Conclusion

Wild bird surveillance in United States has been performed annually during September and November, which is a migratory season and only fecal or cloacal swabs are traditionally collected. After HPAI H5 clade 2.3.4.4 was detected in December 2014, USDA/APHIS wildlife services responded by enhancing surveillance in Pacific Flyway and detected more viruses. Furthermore, multiple introductions of this clade of viruses cannot be ruled out. Therefore, there is a need for more comprehensive wild bird surveillance effort to address a complex wild-domestic animal-human interface to capture variations in subtype specific organ predilection and varying patterns of shedding by different host species.

## Additional file

Additional file 1: Alignment and phylogeny of PB2 segment sequences also captures the 3 stages in evolution amon the outbreak isolates. (ZIP 2258 kb)
