# Supplementary figures and images for: Time-space analysis of highly pathogenic avian influenza H5N2 outbreak in the US
Source: Virol J. 2016 Aug 30;13(1):147. doi: 10.1186/s12985-016-0605-4 (PMC5006563; doi:10.1186/s12985-016-0605-4)

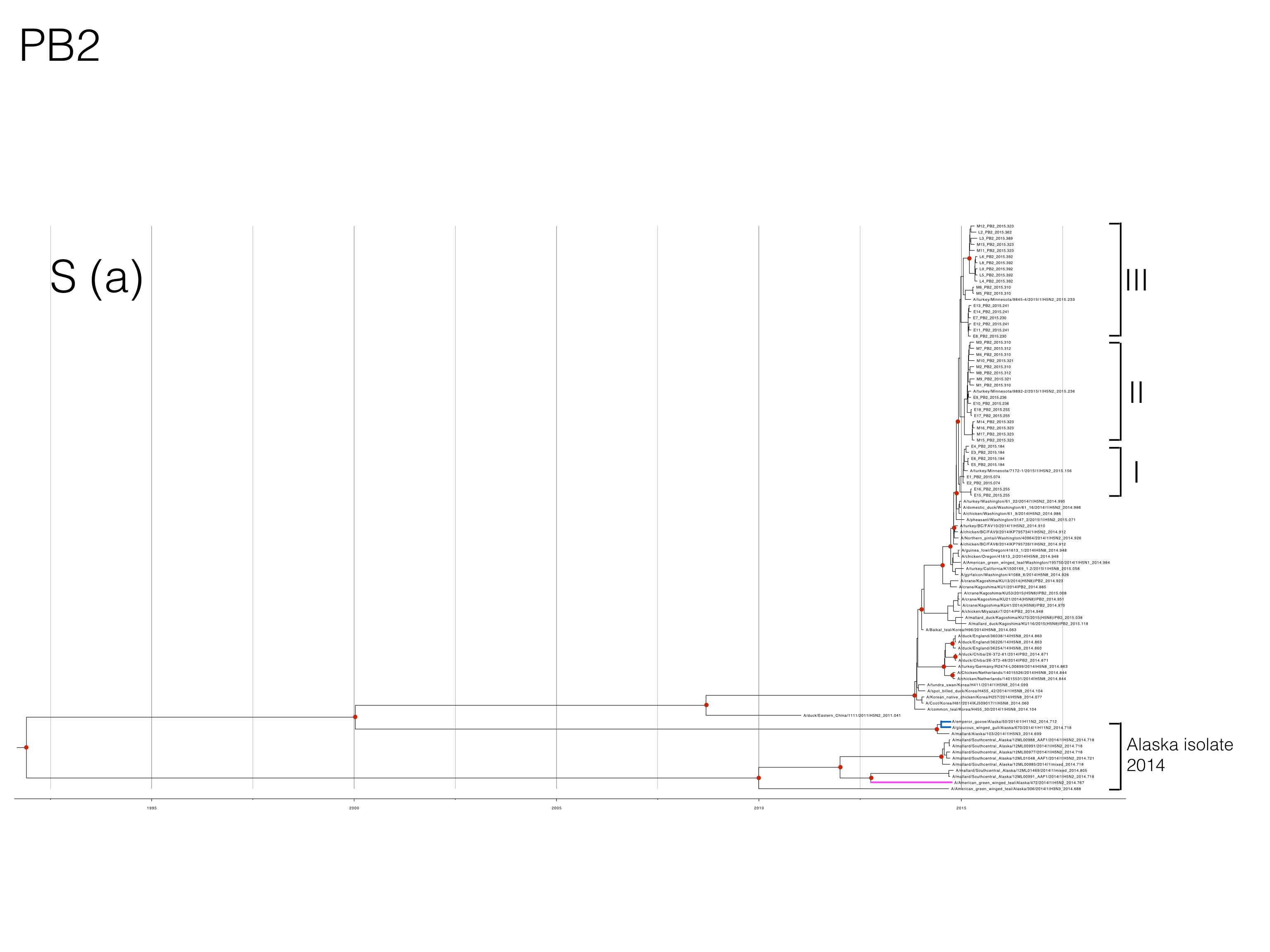

Supplement: Additional file 1: — Alignment and phylogeny of PB2 segment sequences also captures the 3 stages in evolution amon the outbreak isolates. (ZIP 2258 kb) [file 12985_2016_605_MOESM1_ESM.zip › S (a).tiff]

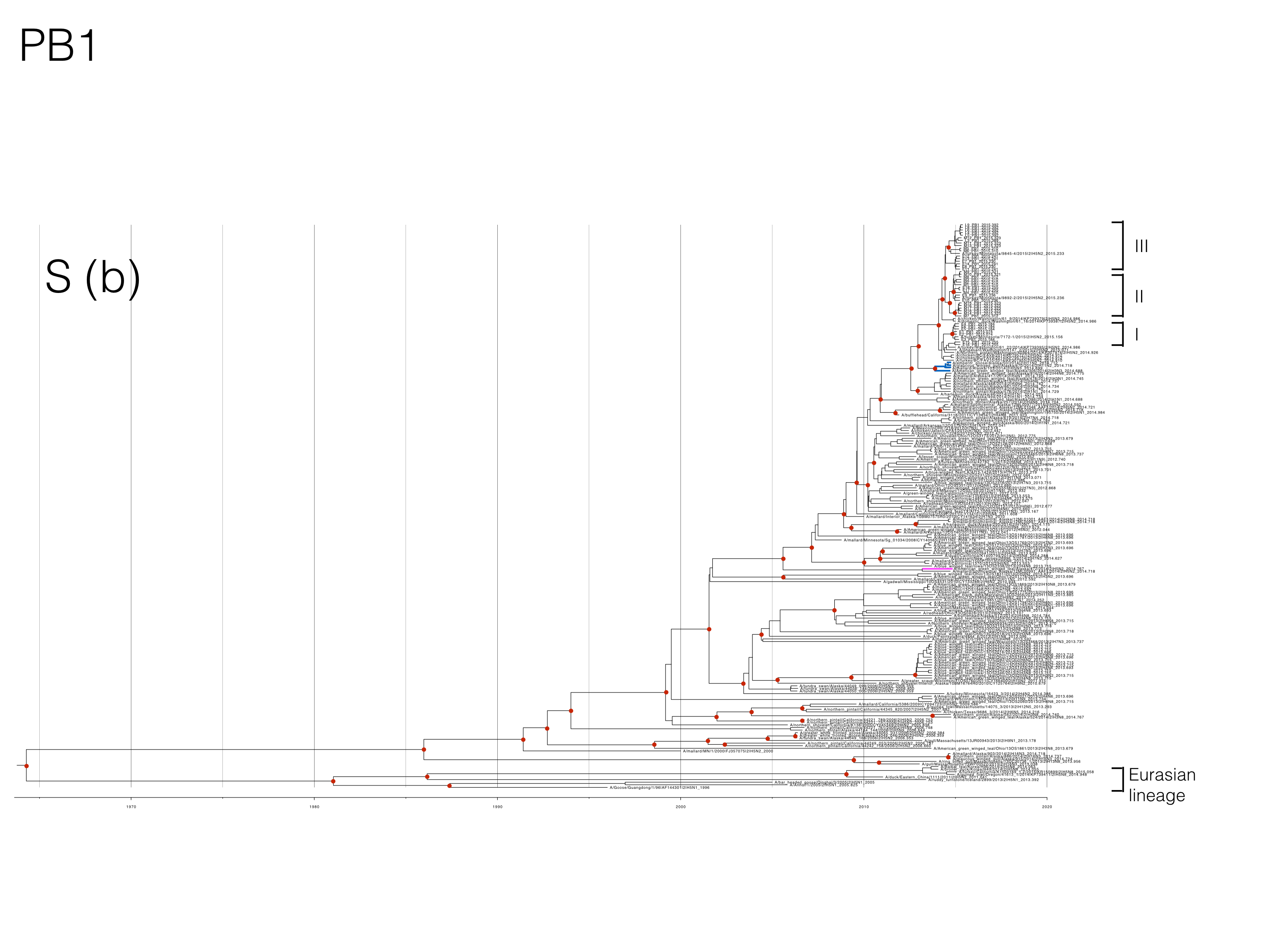

Supplement: Additional file 1: — Alignment and phylogeny of PB2 segment sequences also captures the 3 stages in evolution amon the outbreak isolates. (ZIP 2258 kb) [file 12985_2016_605_MOESM1_ESM.zip › S (b).tiff]

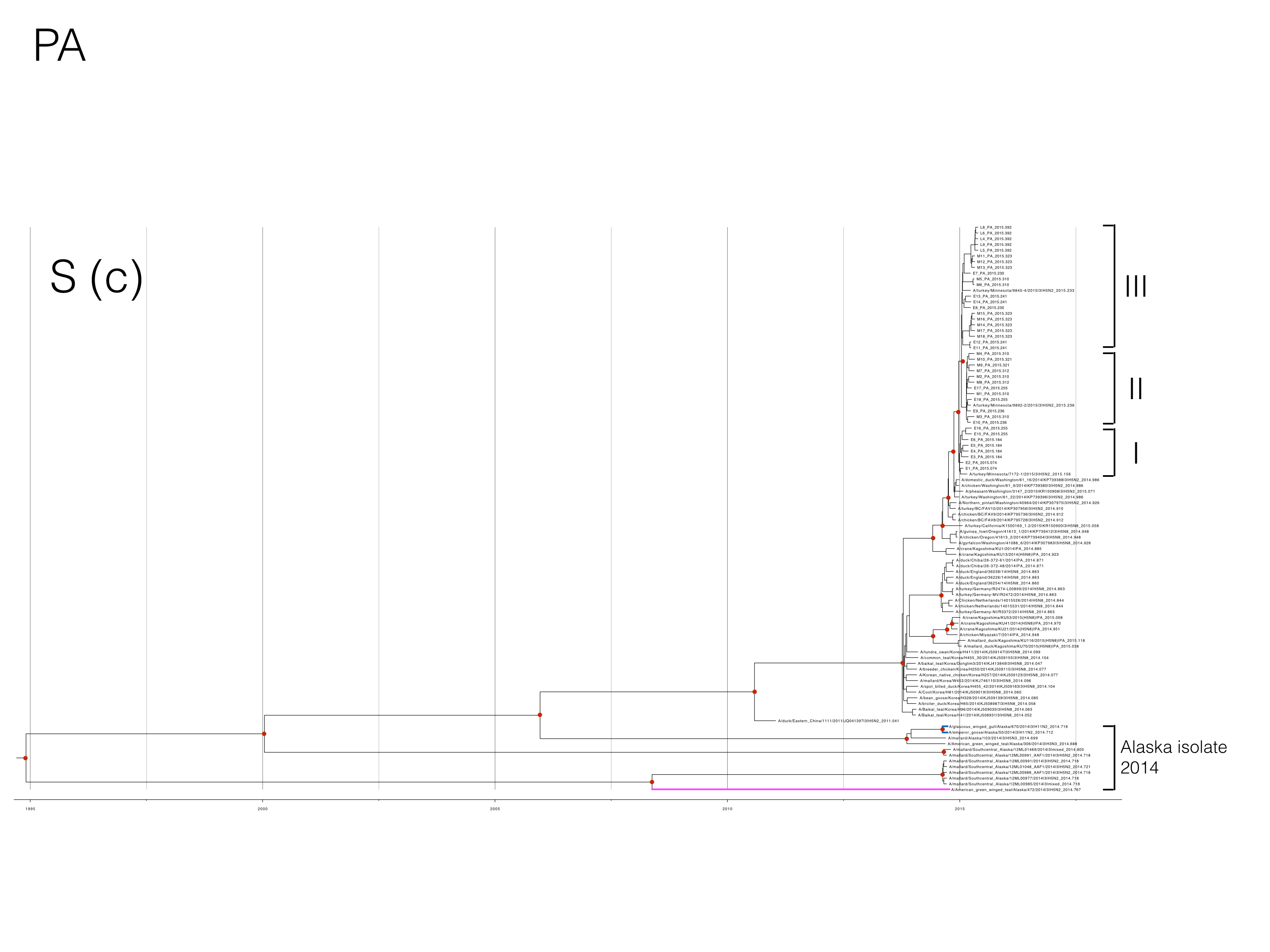

Supplement: Additional file 1: — Alignment and phylogeny of PB2 segment sequences also captures the 3 stages in evolution amon the outbreak isolates. (ZIP 2258 kb) [file 12985_2016_605_MOESM1_ESM.zip › S (c).tiff]

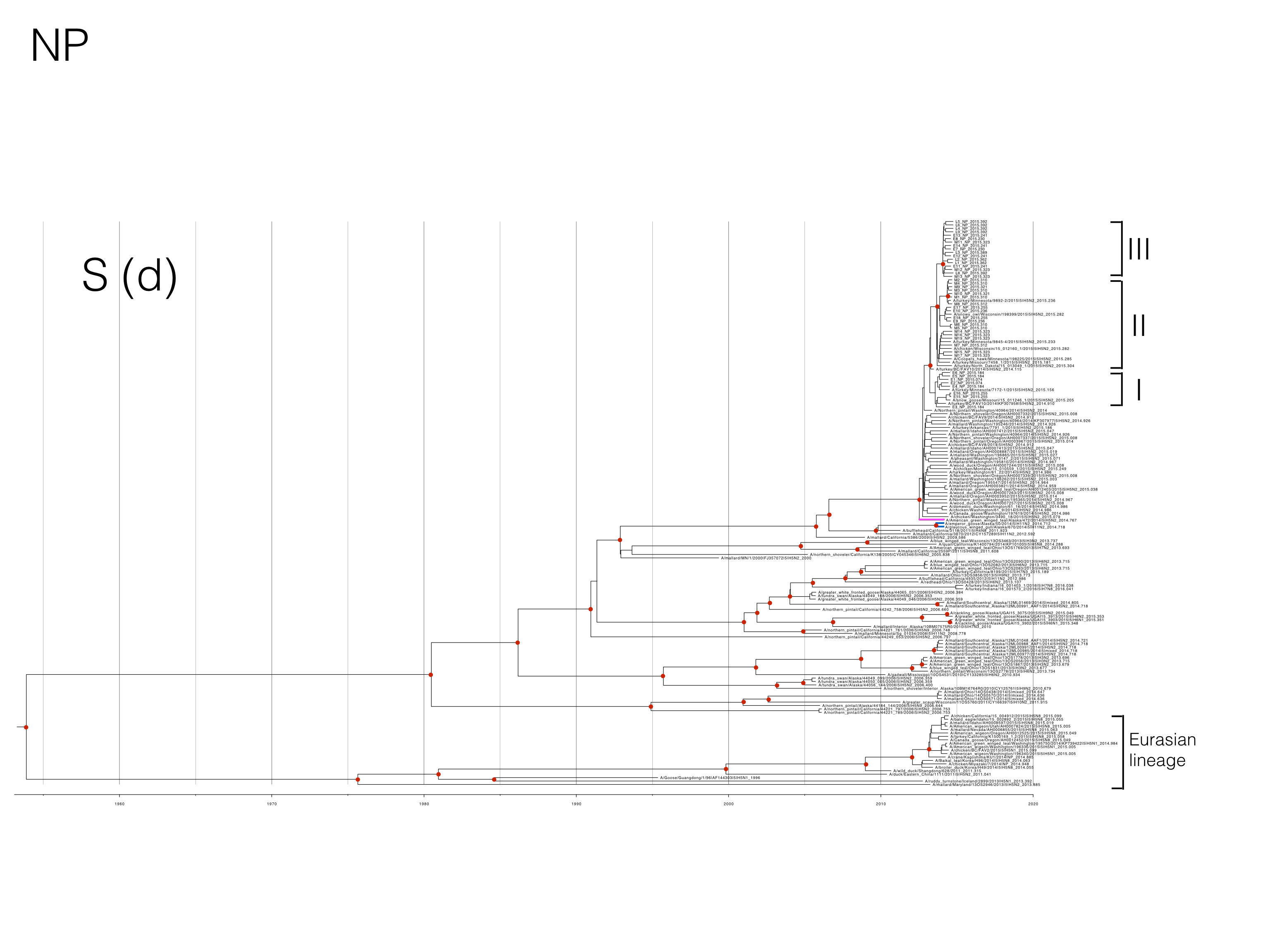

Supplement: Additional file 1: — Alignment and phylogeny of PB2 segment sequences also captures the 3 stages in evolution amon the outbreak isolates. (ZIP 2258 kb) [file 12985_2016_605_MOESM1_ESM.zip › S (d).tiff]

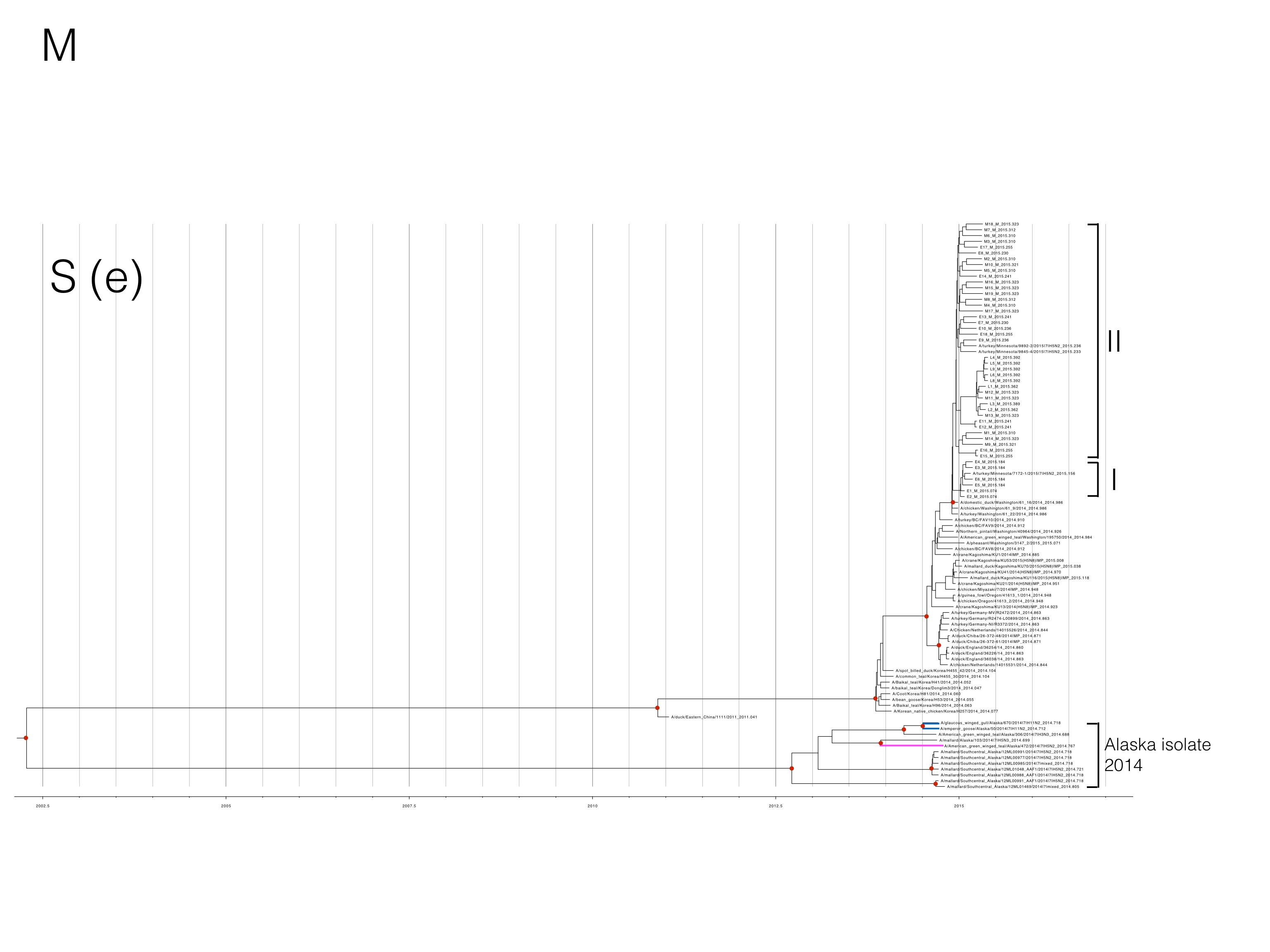

Supplement: Additional file 1: — Alignment and phylogeny of PB2 segment sequences also captures the 3 stages in evolution amon the outbreak isolates. (ZIP 2258 kb) [file 12985_2016_605_MOESM1_ESM.zip › S (e).tiff]
